# Supplementary material for: A model of the cerebellum generates gait adaptations in a reflex-based neuromusculoskeletal model during split-belt walking
Source: bioRxiv. 2025 Nov 24:2024.12.12.628122. Preprint. [Version 3] doi: 10.1101/2024.12.12.628122 (PMC12704001; doi:10.1101/2024.12.12.628122)
Supplement: Supplement 2 — • S4 File pdf. Details on the spinal control laws and controller parameters. [file media-2.pdf]

S4 - Spinal controller

Spinal control equations

The spinal controller is a reflex controller, originally proposed by Geyer and Herr [1], and has been frequently used in other predictive simulation studies [2–6]. In this controller, muscle excitations u are generated from muscle length and force feedback, a proportional-derivative (PD) controller, and constant feedforward signals, as described by the following equations [2]:

Muscle length feedback:

$$u_L = \max(0, K_L(l(t - t_D) - l_0)) \tag{1}$$

Muscle force feedback:

$$u_F = K_F F(t - t_D) \tag{2}$$

PD balance controller:

$$u_{PD} = K_P(\theta(t - t_D) - \theta_0) + K_V(\dot{\theta}(t - t_D)) \tag{3}$$

Feedforward stimulation:

$$u_C = K_C \tag{4}$$

In the equations above, l and f are the normalized muscle length and force, θ and $\dot{\theta}$ are the pelvis tilt and velocity, and t_D is a time delay. The controller gains K_L, K_F, K_P, K_V and K_C are free parameters and whose values are determined during the optimization, as are the length offset of the stretch response l_0 and the desired pelvis tilt angle θ_0 during swing. Mathematically, for the pelvis tilt, it makes no difference whether the desired angle is prescribed or treated as an optimization variable, since the optimized gain K_p can compensate for any fixed choice of the desired angle.

Free controller parameters

For our spinal controller, the above equations, together with the phase-dependent controller depicted in Figure 3 of the main manuscript, lead to the following 31 free spinal control parameters per leg:

	Stance			Swing	
	ES	MS	PS	SW	LP
**HAM**	K_P, K_V, K_C			K_F	
**GLU**	K_P, K_V, K_C		K_C	K_F	
**IL**	K_P, K_V, K_C		K_L, L_0	K_L, L_0, K_P, K_V, θ_0,$K_{L(HAM),}L_{0(HAM)}$	
**VAS**	K_F, K_C, φ				
**GAS**	K_F, K_C				
**SOL**	K_F, K_C				
**TA**	K_L, L_0 $K_{F(SOL)}$				

Figure 1: **Spinal controller parameters for one leg** The parameter values are found during the optimization. To prevent knee overextension, the vastus reflex is only active when knee flexion is above a certain threshold φ, which is also optimized. The same parameters are found for the other leg, resulting in 62 optimization variables.

References

[1] Geyer H, Herr H. A muscle-reflex model that encodes principles of legged mechanics produces human walking dynamics and muscle activities. IEEE transactions on neural systems and rehabilitation engineering: a publication of the IEEE Engineering in Medicine and Biology Society. 2010;18(3):263–273. doi:10.1109/TNSRE.2010.2047592.

[2] Ong CF, Geijtenbeek T, Hicks JL, Delp SL. Predicting gait adaptations due to ankle plantarflexor muscle weakness and contracture using physics-based musculoskeletal simulations. PLoS Computational Biology. 2019;15(10):e1006993. doi:10.1371/journal.pcbi.1006993.

[3] Di Russo A, Stanev D, Armand S, Ijspeert A. Sensory modulation of gait characteristics in human locomotion: A neuromusculoskeletal modeling study. PLoS computational biology. 2021;17(5):e1008594. doi:10.1371/journal.pcbi.1008594.

[4] van der Kruk E, Geijtenbeek T. A planar neuromuscular controller to simulate compensation strategies in the sit-to-walk movement. PLoS one. 2024;19(6):e0305328. doi:10.1371/journal.pone.0305328.

[5] Veerkamp K, Waterval NFJ, Geijtenbeek T, Carty CP, Lloyd DG, Harlaar J, et al.

Evaluating cost function criteria in predicting healthy gait. Journal of Biomechanics. 2021;123:110530. doi:10.1016/j.jbiomech.2021.110530.

[6] Kiss B, Waterval NF, van der Krogt MM, Brehm MA, Geijtenbeek T, Harlaar J, et al. Minimization of metabolic cost of transport predicts changes in gait mechanics over a range of ankle-foot orthosis stiffnesses in individuals with bilateral plantar flexor weakness. Frontiers in Bioengineering and Biotechnology. 2024;12:1369507. doi:10.3389/fbioe.2024.1369507.
