# Supplementary material for: A model of the cerebellum generates gait adaptations in a reflex-based neuromusculoskeletal model during split-belt walking
Source: bioRxiv. 2025 Nov 24:2024.12.12.628122. Preprint. [Version 3] doi: 10.1101/2024.12.12.628122 (PMC12704001; doi:10.1101/2024.12.12.628122)
Supplement: Supplement 4 — • S6 File pdf. Additional kinematic results and muscle activation patterns. [file media-4.pdf]

S6 - Additional Results

Kinematics - additional results

Joint	Leg	Phase	RMSE (°)	NCC
Hip	Fast	EA	8.26	0.99
		LA	7.91	0.99
	Slow	EA	13.57	0.86
		LA	14.93	0.77
Knee	Fast	EA	6.48	0.97
		LA	6.92	0.96
	Slow	EA	10.80	0.93
		LA	13.57	0.81
Ankle	Fast	EA	10.40	0.12
		LA	11.68	-0.03
	Slow	EA	6.17	0.75
		LA	5.34	0.70

Table 1: **Root mean squared error (RMSE) and normalized cross correlation (NCC) between the mean simulated data and experimental data from our representative participant.** RMSE compares the absolute values of the mean trajectories, while NCC is a measure of shape similarity.

Muscle activations - additional results

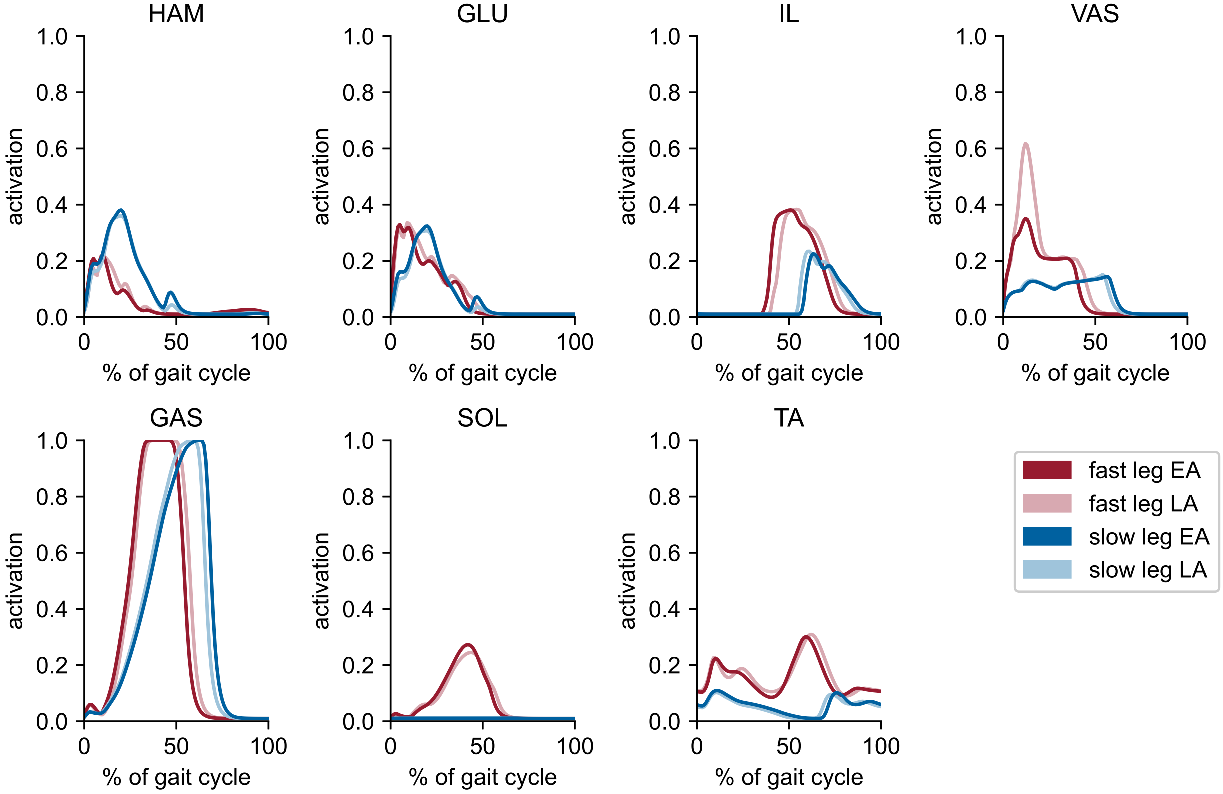

Figure 1: **Muscle activation patterns over time** The trajectories show the average across the 10 simulations with cerebellum.
